# Influence Factors Analyses of PICC‐Related Bloodstream Infection, PICC‐Related Venous Thrombosis, and Infected Puncture Site and Their Influence on Cancer Patients' Death: A Retrospective Cross‐Sectional Study

**DOI:** 10.1002/cam4.70841

**Published:** 2025-04-02

**Authors:** Guizhen Weng, Xiaolan Wu, Suhui Zheng

**Affiliations:** ^1^ Department of Oncology Nursing Fujian Medical University Union Hospital Fuzhou Fujian China; ^2^ School of Nursing, Fujian Medical University Fuzhou Fujian China; ^3^ The Vascular Access Care Centre, Fujian Medical University Union Hospital Fuzhou Fujian China

**Keywords:** nursing, oncology, peripherally inserted central catheter

## Abstract

**Purpose:**

This study aimed to analyze the influence factors affecting catheter‐related bloodstream infection, catheter‐related thrombosis and catheter‐related local complication, respectively, and to explore the relationship among them, as well as the influence of catheter‐related bloodstream infection, catheter‐related thrombosis and catheter‐related local complication on cancer patients' death.

**Methods:**

The clinical data of 605 patients with solid tumors who underwent PICC from March 2019 to January 2020 in a Chinese hospital were retrospectively analyzed. Chi‐square test and logistic regression analyses were used to examine the influence factors affecting catheterrelated bloodstream infection, catheter‐related thrombosis and catheterrelated local complication, respectively, and their influence on cancer patients' death during the follow‐up period.

**Results:**

The results of logistic regression analyses showed that gender, PICC vascular, PICC tip condition, whether anticoagulant therapy and thrombus location were influence factors of catheter‐related bloodstream infection (*p* < 0.05), however, only whether anticoagulant therapy was risk factors of catheter‐related thrombosis [odds ratio (OR) = 7.549, 95% confidence interval (CI): 2.9–19.652, *p* < 0.05]. In addition, surgical history, PICC tip condition and thrombus location were influence factors of catheter‐related local complication (*p* < 0.05). Furthermore, the results showed that after adjusted by all variables, only catheter‐related bloodstream infection was risk factor of cancer patients' death (OR = 11.231, 95% CI: 3.23–39.053, *p* < 0.05). However, catheter‐related thrombosis (OR = 0.793, 95% CI: 0.308–2.043, *p* > 0.05) and catheter‐related local complication (OR = 1.815, 95% CI: 0.715–4.609, *p* > 0.05) were not significantly associated with patients' death.

**Conclusion:**

Overall, the influence factors of catheter‐related bloodstream infection, catheter‐related thrombosis and catheter‐related local complication were significantly different. Moreover, catheter‐related bloodstream infection was the risk factor of cancer patients' death. However, catheter‐related thrombosis and catheter‐related local complication were not significantly associated with patients' death.

## 1 Introduction

Peripherally inserted central catheters (PICCs) have been widely used in patients requiring chemotherapy [1], irritating drug infusion, and intravenous nutrition therapy [2]. For patients requiring long‐term intravenous infusion, PICC has the advantages of reducing the number of punctures and reducing the irritation of high‐concentration drugs to blood vessels because of its ability to remain in the patient's body for a long time [3, 4]. However, with the extension of indwelling time and improper catheter maintenance, the incidence of catheter‐related complications increases, such as catheter‐related bloodstream infection (CRBSI) [5, 6], catheter‐related thrombosis (CRT) [7, 8], and catheter‐related local complication (CRLC) [9]. Among them, CRBSI is an important factor leading to the increased mortality for patients with PICC [10]. Moreover, these PICC‐related complications often lead to an increased unplanned extubation rate and aggravate patients' economic and mental burdens for patients with malignant tumors. In addition, if these complications are not found and treated in time, they may aggravate patients' condition or even lead to patients' death [11]. The majority of studies have focused on exploring risk factors of CRBSI and CRT; however, there are few studies to find influence factors of CRLC [12, 13, 14, 15]. Moreover, few studies reported the potential relationship between CRBSI, CRT, and CRLC, as well as their influence on cancer patients' death. This study aimed to analyze the influencing factors affecting CRBSI, CRT, and CRLC, respectively, and to explore the relationship among CRBSI, CRT, and CRLC, as well as the influence of CRBSI, CRT, and CRLC on cancer patients' death.

## 2 Materials and Methods

### 2.1 Study Design and Participants

All patients with solid tumors who received chemotherapy via a PICC line at Fujian Medical University Union Hospital from March 2019 to January 2020 were included. After the completion of PICC implantation in cancer patients, regular dressing maintenance was continued by the PICC team in the department of medical oncology. Regular dressing maintenance was performed in other local hospitals for patients discharged from the hospital in‐between chemotherapy process.

The participants inclusion criteria were as follows: (1) age more than 18 years old; (2) PICC catheterization under the order and PICC catheterization for more than 3 months; (3) participants without language, cognitive, and intellectual dysfunction; and (4) patients' informed consent. Meanwhile, the exclusion criteria included patients (1) with obvious bleeding tendency; (2) with mental diseases; (3) with heart, lung, liver, kidney, and other important organ dysfuction; (4) with incomplete clinical data; and (5) with pregnancy or lactation period.

The sample size of this study was calculated based on the calculation of 5 times to 10 times the number of variables (15 variables for the influence factors affecting CRBSI, CRT, and CRLC, respectively, besides nine variables for the influence of CRBSI, CRT, and CRLC on cancer patients' death); the sample size of this study could be less than or equal to 150 cases. However, due to the unpredictable number of cancer patients' death during the length of follow‐up and the large source of cancer patients with PICC, the sample size in this study was planned to be more than 300 cases.

### 2.2 Ethical Considerations

This study had been approved by the Ethics Committee of research hospital (Approval No. 2023KY147). The study was conducted in accordance with ethical procedures. All ethical considerations followed by researchers aimed to protect the participants, and all participant data were anonymized. Researchers had the responsibility to make respondents fully understand the purpose, methods, and possible harm of this study. Respondents volunteered to participate in this study, and all participants provided written informed consent when they received chemotherapy via a PICC line during March 2019 to January 2020. Meanwhile, oral informed consent of participants was also received when the telephone follow‐up was conducted to collect the outcomes of cancer patients during March 2024 to September 2024.

### 2.3 Observation Indicators and Evaluation Criteria

Relevant data of patient characteristics were collected. Besides, observational indicators of this study included CRBSI, CRT, and CRLC, as well as patients' death. CRBSIs were defined according to modified criteria of the National Nosocomial Infections Surveillance System of the CDC of Atlanta, USA [16]. CRT was defined as an occlusive or nonocclusive filling defect in the deep vein of the catheter access. It includes brachial, axillary, subclavian, internal jugular, and brachiocephalic veins on the same side of PICC. CRT was confirmed by ultrasonography. CRLC refers to local symptoms of skin tissue at the site of PICC implantation, including local infection, rash, catheter expulsion, and occlusion.

### 2.4 Data Collection

Participants characteristics were collected from the medical system or follow‐up record, including age, gender, body mass index (BMI), cancer type, surgical history, PICC site, PICC vascular, PICC tip condition, Neutrophil count during PICC insertion, platelet (PLT) count during PICC insertion, PICC tip position in chest radiography, PICC dressing maintenance hospital, and comorbidities. Moreover, the research team followed patients weekly to confirm whether PICC‐related complications including CRBSI, CRT, and CRLC occurred until catheter removal with the doctor's advice or patients' death. The follow‐up period of this study was from March 2019 to March 2024. Enoxaparin sodium injection, as the anticoagulant therapy in this study, was used to prevent thrombosis via hypodermic injection once each day with the dosage of 5000 IU when cancer patients received a surgery or had a high level of D‐dimer or had a thromboembolic disease.

### 2.5 Statistical Analysis

The qualitative data were described by absolute value (Number, *N*) and percentage (%). Chi‐square test was used to compare the characteristics of patients and to explore the relationship between CRBSI, CRT, and CRLC. Logistic regression analyses, in which all baseline characteristics of patients were included for analysis, were used to examine the influencing factors affecting CRBSI, CRT, and CRLC, respectively. Besides, a logistic regression analysis was also performed to assess the influence of CRBSI, CRT, and CRLC on cancer patients' death during the follow‐up period. In this logistic analysis model, all baseline characteristics of whether patients died were used as adjusted variables, with CRBSI, CRT, and CRLC as independent variables, and whether patients died as the dependent variable. The odds ratio (OR) and 95% confidence interval (CI) were estimated for the independent variables of the regression analyses. All statistical tests were two‐sided; *p* < 0.05 was considered statistically significant, and statistical analyses were performed using SPSS 25.0 software.

## 3 Results

A total of 605 tumor patients with complete data who underwent PICC insertion were included in this study. There were 56.0% (339 of 605) of patients' aged less than 60. More than half of the patients were female, and the majority of them had a BMI less than 25.

Among them, the incidence of CRBSI, CRT, and CRLC was 2.3% (14 of 605), 11.7% (71 of 605), and 8.4% (51 of 605), respectively. All patient characteristics are shown in Table 1.

**TABLE 1:** Baseline characteristics of patients.

Variable	All patients (*n* = 605)	Patients with CRBSI (*n* = 14)	Patients without CRBSI (*n* = 591)	*p*	Patients with CRT (*n* = 71)	Patients without CRT (*n* = 534)	*p*	Patients with CRLC (*n* = 51)	Patients without CRLC (*n* = 554)	*p*
Age (years)										
< 60	339	10 (71.4%)	329 (55.7%)	0.240	42 (52.2%)	297 (55.6%)	0.573	31 (60.8%)	308 (55.6%)	0.475
≥ 60	266	4 (28.6%)	262 (44.3%)		29 (48.8%)	237 (44.4%)		20 (39.2%)	246 (44.4%)	
Gender										
Male	337	5 (35.7%)	332 (56.2%)	0.128	49 (69.0%)	288 (53.9%)	0.016	27 (52.9%)	310 (56.0%)	0.678
Female	268	9 (64.3%)	259 (43.8%)		22 (31.0%)	246 (46.1%)		24 (47.1%)	244 (44.0%)	
BMI										
< 25	478	11 (78.6%)	467 (79.0%)	0.968	59 (83.1%)	419 (78.5%)	0.368	39 (76.5%)	439 (79.2%)	0.642
≥ 25	127	3 (21.4%)	124 (21.0%)		12 (16.9%)	115 (21.5%)		12 (23.5%)	115 (20.8%)	
Cancer type										
Lung cancer	84	0	84 (14.2%)	0.315	6 (8.5%)	78 (14.6%)	0.442	5 (9.8%)	79 (14.3%)	0.457
Gastric cancer	90	5 (35.7%)	85 (14.4%)		12 (16.9%)	78 (14.6%)		4 (7.8%)	86 (15.5%)	
Colorectal cancer	241	7 (50.0%)	234 (39.6%)		35 (49.3%)	206 (38.6%)		28 (54.9%)	213 (38.4%)	
Breast cancer or ovarian cancer	72	1 (7.1%)	71 (12.0%)		6 (8.5%)	66 (12.4%)		4 (7.8%)	68 (12.3%)	
Esophageal cancer	29	0	29 (4.9%)		5 (7.0%)	24 (4.5%)		2 (3.9%)	27 (4.9%)	
Liver cancer	18	0	8 (1.4%)		1 (1.4%)	7 (1.3%)		1 (2.0%)	7 (1.3%)	
Head cancer	12	0	12 (2.0%)		1 (1.4%)	11 (2.1%)		1 (2.0%)	11 (2.0%)	
Other cancer	69	1 (7.1%)	68 (11.5%)		5 (7.0%)	54 (12.0%)		6 (11.8%)	63 (11.4%)	
Surgical history										
Yes	402	6 (42.9%)	396 (67.5%)	0.053	14 (87.5%)	389 (66.6%)	0.079	25 (49.0%)	377 (68.5%)	0.005
No	199	8 (57.1%)	191 (32.5%)		2 (12.5%)	195 (33.4%)		26 (51.0%)	173 (31.5%)	
PICC site										
Left hand	265	4 (28.6%)	261 (44.2%)	0.245	33 (46.5%)	232 (43.4%)	0.628	25 (49.0%)	240 (43.3%)	0.433
Right hand	340	10 (71.4%)	330 (55.8%)		38 (53.5%)	302 (56.6%)		26 (51.0%)	314 (56.7%)	
PICC vascular										
Basilic vein	442	4 (28.6%)	438 (74.1%)	0.001	55 (77.5%)	387 (72.5%)	0.642	37 (72.5%)	405 (73.1%)	0.950
Brachial vein	162	10 (71.4%)	152 (25.7%)		16 (22.5%)	146 (27.3%)		14 (27.5%)	148 (26.7%)	
Cephalic vein	1	0	1 (0.2%)		0	1 (0.2%)		0	1 (0.2%)	
PICC tip condition										
Closure	594	11 (78.6%)	583 (98.6%)	0.000	69 (97.2%)	525 (98.3%)	0.503	47 (92.2%)	547 (98.7%)	0.001
Open	11	3 (21.4%)	8 (1.4%)		2 (2.8%)	9 (1.7%)		4 (7.8%)	7 (1.3%)	
Neutrophil count										
< 500/cm^3^	10	0	10 (1.7%)	0.624	0	10 (1.9%)	0.245	0	10 (1.8%)	0.333
≥ 500/cm^3^	595	14 (100%)	581 (98.3%)		71 (100%)	524 (98.1%)		51 (100.0%)	544 (98.2%)	
PLT count										
< 20,000	17	0	17 (2.9%)	0.520	1 (1.4%)	16 (3.0%)	0.447	0	17 (3.1%)	0.204
≥ 20,000	588	14 (100%)	574 (97.1%)		70 (98.6%)	518 (97.0%)		51 (100.0%)	537 (96.9%)	
PICC tip position in chest radiography										
T4‐5	8	0	8 (1.4%)	0.058	0	8 (1.5%)	0.496	1 (2.0%)	7 (1.3%)	0.122
T6‐7	377	13 (92.9%)	364 (61.6%)		47 (66.2%)	330 (61.8%)		25 (49.0%)	352 (63.5%)	
T8‐9	220	1 (7.1%)	219 (37.1%)		24 (33.8%)	196 (36.7%)		25 (49.0%)	195 (35.2%)	
PICC dressing maintenance hospital										
FJMU	181	3 (21.4%)	178 (30.1%)	0.483	17 (23.9%)	164 (30.7%)	0.242	21 (41.2%)	160 (28.9%)	0.066
Other	424	11 (28.6%)	413 (69.9%)		54 (76.1%)	370 (69.3%)		30 (58.8%)	394 (71.1%)	
Comorbidities										
None	443	10 (71.4%)	433 (73.3%)	0.858	54 (76.1%)	389 (72.8%)	0.886	35 (68.6%)	408 (73.6%)	0.481
Hypertension	95	3 (21.4%)	92 (15.6%)		11 (15.5%)	84 (15.7%)		8 (15.7%)	87 (15.7%)	
Diabetes	30	1 (7.1%)	29 (4.9%)		3 (4.2%)	27 (5.1%)		5 (9.8%)	25 (4.5%)	
Coronary heart disease	6	0	6 (1.0%)		1 (1.4%)	5 (0.9%)		1 (2.0%)	5 (0.9%)	
Hypertension, diabetes mellitus, and coronary artery disease	31	0	31 (5.2%)		2 (2.8%)	29 (5.4%)		2 (3.9%)	29 (5.2%)	
Whether anticoagulant therapy										
Yes	95	7 (50.0%)	88 (14.9%)	0.000	32 (45.1%)	63 (11.8%)	0.000	12 (23.5%)	83 (15.0%)	0.108
No	510	7 (50.0%)	503 (85.1%)		39 (54.9%)	471 (88.2%)		39 (76.5%)	417 (85.0%)	
Thrombus location										
Brachial vein	11	3 (21.4%)	8 (1.4%)	0.000	19 (26.8%)	0	0.000	3 (5.9%)	8 (1.4%)	0.036
Axillary vein	25	1 (7.1%)	24 (4.1%)		11 (15.5%)	0		4 (7.8%)	21 (3.8%)	
Venae subclavia	16	0	16 (2.7%)		25 (35.2%)	0		0	16 (2.9%)	
No	553	10 (71.4%)	543 (98.2%)		16 (22.5%)	534 (100.0%)		44 (86.3%)	509 (91.9%)	

Abbreviations: CRBSI, catheter‐related bloodstream infection; CRLC, catheter‐related local complication; CRT, catheter‐related thrombosis; FJMU, Fujian Medical University Union Hospital; PICC, peripherally inserted central venous catheters; PLT, platelet.

Meanwhile, univariate analyses were used by the chi‐square test analyses to explore single factors of CRBSIs, CRT, and CRLC, respectively. The results showed that PICC vascular, PICC tip condition, whether anticoagulant therapy, and thrombus location were single influence factors of CRBSIs (*p <* 0.05). Gender, whether anticoagulant therapy, and thrombus location were single risk factors of CRT (*p <* 0.05). Besides, surgical history, PICC tip condition, and thrombus location were single influence factors of CRLC (*p <* 0.05). All results are shown in Table 1.

The chi‐square test analyses were used to further explore the difference among CRLC, CRBSI, and CRT. The results showed that there were significant differences between patients with/without CRBSI and patients with/without CRT (*p <* 0.05). Only six patients were both with CRBSI and CRT, and 65 participants were without CRBSI but with CRT. Besides, patients with/without CRBSI were significantly different from patients with/without CRLC (*p <* 0.05). There were only 10 patients both with CRLC and CRT; however, a total of 61 participants were only with CRT. Moreover, it showed a significant difference between patients with/without CRLC and patients with/without CRT (*p <* 0.05). Only four cancer patients were both with CRBSI and CRLC, whereas 47 participants were only with CRLC (see Table 2).

**TABLE 2:** Difference analyses of CRLC with CRBSI and CRT.

Variable	Patients with CRT (*n* = 71)	Patients without CRT (*n* = 534)	*p*	Patients with CRLC (*n* = 51)	Patients without CRLC (*n* = 554)	*p*
Patients with CRBSI (*n* = 14)	6	8	0.000	4	10	0.006
Patients without CRBSI (*n* = 591)	65	526		47	554	
Patients with CRLC (*n* = 51)	10	41	0.068	\	\	\
Patients without CRLC (*n* = 554)	61	493		\	\	

Moreover, logistic regression analyses were used to examine the influence factors affecting CRBSI, CRT, and CRLC, respectively. The results showed that gender, PICC vascular, PICC tip condition, whether anticoagulant therapy, and thrombus location were influence factors of CRBSIs (*p <* 0.05). Female had a 7.218 times higher risk of CRBSI compared to that of male (95% CI: 1.362–38.264). Compared with cancer patients with PICC vascular of basilic vein, patients with PICC vascular of brachial vein had a high risk of CRBSI (OR = 8.926, 95% CI: 2.311–34.472). Patients with PICC tip open were 44.351 times higher risk of CRBSI than that of patients with PICC tip closure (95% CI: 6.374–308.592). Patients with thrombus location in axillary vein had an 85.295 times higher risk of CRBSI compared to that of patients with thrombus location in brachial vein (95% CI: 9.714–748.947). The risk of CRBSI in cancer patients receiving the anticoagulant therapy was 4.681 times higher than that of patients not receiving the anticoagulant therapy (95% CI: 1.314–16.68). However, only whether anticoagulant therapy was the risk factor of CRT (*p <* 0.05); the risk of CRT in cancer patients with the anticoagulant therapy was 7.549 times higher than that of patients without the anticoagulant therapy (95% CI: 2.9–19.652). In addition, surgical history, PICC tip condition, and thrombus location were influence factors of CRLC (*p <* 0.05). Compared with patients not having a surgical history, cancer patients with a surgical history had a low risk of CRLC (OR = 0.452, 95% CI: 0.248–0.823). The risk of CRLC in cancer patients with PICC tip open was 4.68 times higher than that of patients with PICC tip closure (95% CI: 1.281–17.103). Cancer patients with thrombus location in axillary vein had a 5.526 times higher risk of CRLC compared to patients with thrombus location in brachial vein (95% CI: 1.368–22.316). All results are shown in Table 3.

**TABLE 3:** Influence factor analyses of CRBSI, CRT, and CRLC, respectively.

Variable	*B*	S.E.	Wald value	*p*	OR	95% CI	
						Lower	Upper
Influence factors of CRBSI							
Female (Ref. Male)	1.977	0.851	5.394	0.02	7.218	1.362	38.264
PICC vascular of basilic vein (Ref.)			10.083	0.006			
PICC vascular of brachial vein	2.189	0.689	10.083	0.001	8.926	2.311	34.472
PICC vascular of cephalic vein	−12.856	40192.97	0	1	0	0	—
PICC tip open (Ref. PICC tip Closure)	3.792	0.99	14.68	0	44.351	6.374	308.592
Thrombus location in brachial vein (Ref.)			16.089	0.001			
Thrombus location in axillary vein	4.446	1.108	16.088	0	85.295	9.714	748.947
Thrombus location in venae subclavia	0.534	1.317	0.164	0.685	1.705	0.129	22.512
No thrombus	−17.309	8548.272	0	0.998	0	0	—
Anticoagulant therapy (Ref. No anticoagulant therapy)	1.544	0.648	5.669	0.017	4.681	1.314	16.68
Constant	−8.347	1.415	34.813	0	0		
Influence factors of CRT							
Anticoagulant therapy (Ref. No anticoagulant therapy)	2.021	0.488	17.148	0	7.549	2.9	19.652
Constant	−3.546	0.347	104.6	0	0.029		
Influence factors of CRLC							
Surgical history (Ref. No surgical history)	−0.794	0.306	6.754	0.009	0.452	0.248	0.823
PICC tip open (Ref. PICC tip Closure)	1.543	0.661	5.448	0.02	4.68	1.281	17.103
Thrombus location in brachial vein (Ref.)			7.406	0.06			
Thrombus location in axillary vein	1.709	0.712	5.762	0.016	5.526	1.368	22.316
Thrombus location in venae subclavia	0.844	0.587	2.069	0.15	2.326	0.736	7.35
No thrombus	−18.596	9983.351	0	0.999	0	0	—
Constant	−1.279	1.103	1.345	0.246	0.278		

In addition, baseline characteristics of whether patients died during the follow‐up period are also shown in Table 4. There were 9.3% (56 of 605) of patients who died during the follow‐up period. Only CRBSI was the single risk factor for whether patients died during the follow‐up period (*p <* 0.05), whereas there were significant differences between age, gender, BMI, cancer type, surgical history, comorbidities and whether patients died during the follow‐up period (*p* > 0.05).

**TABLE 4:** Baseline characteristics of whether patients died during the follow‐up period.

Variable	All patients (*n* = 605)	Patients died during the follow‐up period (*n* = 56)	Patients survived during the follow‐up period (*n* = 549)	*p*
Age (years)				
< 60	339	27 (48.2%)	312 (56.8%)	0.216
≥ 60	266	29 (51.8%)	237 (43.2%)	
Gender				
Male	337	29 (51.8%)	308 (56.1%)	0.536
Female	268	27 (48.2%)	241 (43.9%)	
BMI				
< 25	478	45 (80.4%)	433 (78.9%)	0.795
≥ 25	127	11 (19.6%)	116 (21.1%)	
Cancer Type				
Lung cancer	84	7 (12.5%)	77 (14.0%)	0.257
Gastric cancer	90	12 (21.4%)	78 (14.2%)	
Colorectal cancer	241	14 (25.0%)	227 (41.3%)	
Breast cancer or ovarian cancer	72	9 (16.1%)	63 (11.5%)	
Esophageal cancer	29	4 (7.1%)	25 (4.6%)	
Liver cancer	18	0	8 (1.5%)	
Head cancer	12	2 (3.6%)	10 (1.8%)	
Other cancer	69	8 (14.3%)	61 (11.1%)	
Surgical history				
Yes	402	38 (69.1%)	364 (66.7%)	0.716
No	199	17 (30.9%)	182 (33.3%)	
Comorbidities				
None	443	39 (73.2%)	404 (73.6%)	0.950
Hypertension	95	10 (17.9%)	85 (15.5%)	
Diabetes	30	3 (5.4%)	27 (4.9%)	
Coronary heart disease	6	1 (1.8%)	5 (0.9%)	
Hypertension, diabetes mellitus, and coronary artery disease	31	3 (5.4%)	28 (5.1%)	
Whether patients with CRBSI				
Yes	14	6 (42.9%)	8 (57.1%)	0.000
No	591	50 (8.5%)	541 (91.5%)	
Whether patients with CRT				
Yes	71	7 (9.9%)	64 (90.1%)	0.852
No	534	49 (9.2%)	485 (90.8%)	
Whether patients with CRLC				
Yes	51	7 (13.7%)	44 (86.3%)	0.250
No	554	49 (8.8%)	505 (91.2%)	

Furthermore, a logistic regression analysis was used to examine the influence of CRBSI, CRT, and CRLC on cancer patients' death during the follow‐up period (from March 2019 to March 2024). The results showed that after adjusted by all variables, only CRBSI was the risk factor of cancer patients' death (OR = 11.231, 95% CI: 3.23–39.053, *p <* 0.05). However, CRT (OR = 0.793, 95% CI: 0.308–2.043, *p* > 0.05) and CRLC (OR = 1.815, 95% CI: 0.715–4.609, *p* > 0.05) were not significantly associated with patients' death. All results were shown in Table 5.

**TABLE 5:** Influence factors including CRBSI, CRT, and CRLC of whether patients died during the follow‐up period.

Variable	*B*	S.E.	Wald value	*p*	Exp(B)	95% CI	
						Lower	Upper
Age ≥ 60 (Ref. Age < 60)	0.515	0.301	2.929	0.087	1.674	0.928	3.019
Female (Ref. Male)	0.005	0.341	0	0.987	1.005	0.516	1.96
BMI ≥ 25 (Ref. BMI < 25)	−0.214	0.373	0.33	0.566	0.807	0.388	1.678
No comorbidities (Ref.)			1.226	0.874			
Hypertension	0.174	0.389	0.2	0.655	1.19	0.555	2.55
Diabetes	0.162	0.658	0.061	0.806	1.176	0.324	4.273
Coronary heart disease	1.12	1.138	0.969	0.325	3.066	0.33	28.521
Hypertension, diabetes mellitus, and coronary artery disease	0.269	0.647	0.173	0.677	1.309	0.368	4.656
Lung cancer (Ref.)			9.16	0.241			
Gastric cancer	0.334	0.523	0.407	0.523	1.397	0.501	3.895
Colorectal cancer	−0.575	0.502	1.311	0.252	0.563	0.211	1.505
Breast cancer or ovarian cancer	0.425	0.593	0.514	0.473	1.53	0.478	4.895
Esophageal cancer	0.726	0.681	1.137	0.286	2.067	0.544	7.854
Liver cancer	−18.986	14067.065	0	0.999	0	0	—
Head cancer	0.99	0.891	1.236	0.266	2.692	0.47	15.429
Other cancer	0.24	0.578	0.173	0.678	1.271	0.41	3.945
Surgical history (Ref. No surgical history)	0.374	0.339	1.217	0.27	1.453	0.748	2.821
Patients with CRBSI (Ref. without CRBSI)	2.419	0.636	14.468	0	11.231	3.23	39.053
Patients with CRT (Ref. without CRT)	−0.232	0.483	0.231	0.631	0.793	0.308	2.043
Patients with CRLC (Ref. without CRLC)	0.596	0.476	1.57	0.21	1.815	0.715	4.609
Constant	−2.989	0.518	33.31	0	0.05		

## 4 Discussion

PICC has the advantages of safety, long indwelling time, not easy to fall off, low cost, and less stimulation to patients with vascular injury and improve the quality of life [17]. PICC is often used in treating patients requiring chemotherapy and continuous intravenous nutrition infusion in clinical practice. Notably, PICC is an invasive procedure that may cause related complications, including CRBSI, CRT, and CRLC [18]. These complications may cause treatment interruption or delay in systemic cancer treatment and even endanger the patient's life in severe cases. Our study found that the incidence of CRBSI, CRT, and CRLC was 2.3% (14 of 605), 11.7% (71 of 605), and 8.4% (51 of 605), respectively. These results were similar to previous studies. Thomsen et al. found that among patients who received medium‐term to long‐term intravenous therapy, the incidence of CRBSI was low, with 0 (0 of 152) in the midline catheters group and 0.7% (1 of 152) in the PICC control group and there was no difference between PICC and midline catheters groups [19]. Wang et al. showed that 11.48% (35 of 305) of lymphoma patients occurred CRT [20].

As a serious complication of PICC [10], CRBSI refers to bacteremia or fungemia accompanied by fever (body temperature > 38°C), chills or hypotension, and other infection manifestations in patients with PICC or within 48 h after withdrawal of PICC, and without another clear source of infection except for catheter infection. Many studies have analyzed the risk factors for CRBSI in patients with PICC. Pongruangporn et al. [15] found that congestive heart failure, intra‐abdominal perforation, clostridium difficile infection, recent chemotherapy, and tracheostomy presence were independent risk factors for CRBSI. In another study, CRBSI was most associated with hospital length of stay, ICU status, and number of device lumens [13]. This study found that patients with/without CRBSI were different from PICC vascular, PICC tip condition, whether anticoagulant therapy, and thrombus location. Meanwhile, gender, PICC vascular, PICC tip condition, whether anticoagulant therapy, and thrombus location were influence factors of CRBSI. Besides, there were significant differences between patients with/without CRBSI and patients with/without CRT, as well as between patients with/without CRBSI and patients with/without CRLC. Almost all catheter‐infected patients have manifestations of fever, and the only manifestation in about one‐third of patients is fever. Usually, the sudden onset of a high fever combined with toxic symptoms suggests possible CRBSI. Confirmation of CRBSI requires either a positive culture from peripheral venous blood for bacteria or fungi by laboratory microbiology or a culture of a pathogenic bacterium of the same species with the same susceptibility result from the catheter segment and peripheral blood. Once CRBSI occurs, removing the PICC tube and performing active anti‐infective treatment is necessary. If it is not treated in time, it may endanger the patient's life.

As the common complication of PICC [21], CRT refers to the process of blood clot formation in the inner wall of the vessel where the catheter is located or the attachment wall of the catheter because of multiple factors such as puncture or catheter damage to the venous vessel and the patient's state after venous catheterization [22]. CRT may lead to deep venous thrombosis and pulmonary embolism, resulting in increased unplanned extubation rates, increasing the economic and mental burden of patients, and in severe cases, death of patients [23]. Our study found that patients with/without CRT were different in gender, whether anticoagulant therapy and thrombus location. And the anticoagulant therapy was the only risk factor of CRT (OR = 7.549, 95% CI: 2.9–19.652). Similarly, Jasti et al. illustrated that a history of venous embolism was a risk factor for CRT formation [14]. Chopra et al. [12] found that PICC patients with a history of venous embolism were more likely to form CRT. Lobo et al. [24] found that a history of venous embolism increased the risk of CRT by 10.33 times. The 10‐year recurrence rate of venous thrombosis was 30%–50% [25], possibly due to different degrees of coagulation–anticoagulation and systemic dysfunction in patients with a history of venous embolism. PICC implantation causes endothelial cell damage, activating the coagulation system and inhibiting the fibrinolytic system. Therefore, in clinical nursing, attention should be paid to the regular evaluation of patients with a history of venous embolism, and accurate preventive and nursing measures should be given to prevent the occurrence of thrombosis.

CRLC includes local infection, rash, catheter expulsion, and occlusion of PICC. PICC is an invasive procedure that increases the chance of bacterial invasion. Bleeding, exudates, and secretions at the puncture site provide a culture medium for the bacteria, increasing the risk of local infection, phlebitis, and fibrin sheath of the catheter. The occurrence of CRLC can lead to an increased risk of CRBSI, increasing the probability of CRT [26]. More studies have focused on finding the risk factors of CRBSI and CRT; however, there are still few studies on the risk factors of CRLC. We found that patients with/without CRLC differed based on surgical history, PICC tip condition, and thrombus location. Moreover, surgical history, PICC tip condition, and thrombus location were influencing factors of CRLC. Besides, there were significant differences between patients with/without CRLC and patients with/without CRBSI, as well as between patients with/without CRLC and patients with/without CRT. The proportion of CRLC occurring in clinical practice is higher than that of CRBSI and CRT. The clinical symptoms of CRLC are more obvious and can be found in time through regular care, so it is particularly important for the maintenance of PICC. For catheterization patients with abnormal puncture sites, timely nursing measures should be taken. Patients with PICC lines should avoid heavy lifting or excessive activity. In case of blood seepage and exudation, patients should seek medical advice promptly and replace the dressing adhesive film.

With the gradual prevalence of PICC use, more and more studies have also found that the use of PICC does not completely save patients from CRBSI [27, 28], which may also be related to factors such as decreased immunity in cancer patients after radiotherapy and chemotherapy. Furthermore, we also found that after adjusted for all variables, only CRBSI was a risk factor of cancer patients' death. However, CRT and CRLC were not significantly associated with patients' death. Significant events impacting healthcare over the last several years have been associated with escalating rates of healthcare‐associated infections. This has resulted in increased efforts to reinstitute well‐established and evidence‐based infection prevention practices, particularly for central line associated bloodstream infections [29]. A previous study focused on the safety and effectiveness of tunneled PICC versus conventional PICC in adult cancer patients, which found that catheter‐related complications are associated with the technique of catheterization [19]. Compared with conventional PICC, tunneled PICC reduced catheter‐related long‐term complications. Tunneled PICC placement provides an alternative catheterization method for cancer patients [30]. Another study also indicated that the implanted vascular access ports achieved safety benefits compared with chemotherapy through PICC. Therefore, the implanted vascular access ports are regarded as safe and effective vascular access for the administration of chemotherapy. When considering economic factors and some key elements, more high‐quality research would help verify these clinical benefits [31]. The implanted port catheters are safer and therefore should be preferred in this setting regardless of catheter dwell time [32].

The strengths of this study lie in its large sample size focusing on influence factors analyses of CRBSI, CRT, and CRLC, and their influence on cancer patients' death. However, there were also some limitations. On the one hand, cancer patients were only selected from one hospital in China because of the time and manpower, which might result in biases. On the another hand, survival rate of cancer patients was an important outcome in this study; however, the length of follow‐up in this study was less than 5 years. That might result in a superficial conclusion of the influence of CRBSI, CRT, and CRLC on cancer patients' death. More high‐quality, multicenter studies that have a longer follow‐up are necessary to generate scientific evidence in the future.

## 5 Conclusion

The PICC technique is gradually and widely used in clinical practice, with a low difficulty coefficient and a high success rate, which is safer than peripheral veins. Overall, the risk factors of CRBSIs, CRT, and CRLC were significantly different. Moreover, CRBSI was a risk factor for cancer patients' death. However, CRT and CRLC were not significantly associated with patients' death.

## 6 Relevance to Clinical Practice

The analysis of the risk factors of PICC‐related complications is helpful for nurses or other health workers to carry out targeted nursing intervention for patients with PICC, effectively preventing the occurrence of PICC‐related complications in cancer patients and reducing the risk of death. In this study, CRBSI was a risk factor for cancer patients' death. Gender, PICC vascular access, PICC tip condition, whether anticoagulant therapy was used, and thrombus location were influencing factors of CRBSIs. Therefore, it is of great clinical significance to analyze the relevant factors related to the occurrence of CRBSI for PICC patients to prevent and treat them. To reduce the incidence of CRBSIs, health workers need to pay more attention and provide nursing care for female patients with PICC and to first select the basilic vein during PICC puncture. Additionally, anticoagulant therapy would not benefit cancer patients with PICC in reducing the incidence of CRBSI. Therefore, health workers should pay attention to adopt the correct care methods to maintain the PICC line.

## Author Contributions

**Guizhen Weng:** conceptualization (equal), data curation (equal), formal analysis (equal), project administration (equal), writing – original draft (equal), writing – review and editing (equal). **Xiaolan Wu:** formal analysis (equal), writing – review and editing (equal). **Suhui Zheng:** formal analysis (equal), writing – review and editing (equal).

## Conflicts of Interest

The authors declare no conflicts of interest.

## Data Availability

The datasets from the current study are available from the corresponding author upon request.
